# Genetics of the ovarian reserve

**DOI:** 10.3389/fgene.2015.00308

**Published:** 2015-10-15

**Authors:** Emanuele Pelosi, Antonino Forabosco, David Schlessinger

**Affiliations:** ^1^Intramural Research Program, National Institute on Aging, National Institutes of Health, Baltimore, MD, USA; ^2^Genomic Research Centre, Cante di Montevecchio Association, Fano, Italy

**Keywords:** ovarian reserve, reproduction, reproductive lifespan, menopause, folliculogenesis

## Abstract

Primordial follicles or non-growing follicles (NGFs) are the functional unit of reproduction, each comprising a single germ cell surrounded by supporting somatic cells. NGFs constitute the ovarian reserve (OR), prerequisite for germ cell ovulation and the continuation of the species. The dynamics of the reserve is determined by the number of NGFs formed and their complex subsequent fates. During the reproductive lifespan, the OR progressively diminishes due to follicle atresia as well as recruitment, maturation, and ovulation. The depletion of the OR is the major determining driver of menopause, which ensues when the number of primordial follicles falls below a threshold of ∼1,000. Therefore, genes and processes involved in follicle dynamics are particularly important to understand the process of menopause, both in the typical reproductive lifespan and in conditions like primary ovarian insufficiency, defined as menopause before age 40. Genes and their variants that affect the timing of menopause thereby provide candidates for diagnosis of and intervention in problems of reproductive lifespan. We review the current knowledge of processes and genes involved in the development of the OR and in the dynamics of ovarian follicles.

## Introduction

The growing trend in all contemporary societies for childbearing later in women’s lives, accompanied by the increasing use of assisted reproduction technology, make the term ovarian reserve (OR) increasingly prominent in medical and scientific literature. Coined more than 25 years ago by Navot et al. (1987), it occurs in at least 1,500 papers currently in PubMed, with an exponentially increasing prevalence. However, the concept of OR is rarely defined, and varies in its usage in different contexts. In current clinical literature OR unambiguously signifies the number and quality of the follicles remaining in an ovary at any given time (Broekmans et al., 2006) or a woman’s reproductive potential as a function of the number and quality of her remaining oocytes (American College of Obstetricians and Gynecologists (ACOG), 2015). By contrast, the research literature considers that the mammalian ovary contains two functional pools: a pool of resting or non-growing follicles (NGFs) and another of growing follicles. A resting follicle is “primordial,” comprised of an oocyte surrounded by a single layer of flattened somatic pre-granulosa cells and arrested in the diplotene/dictyate stage of meiotic prophase I. Such a follicle continues to be quiescent throughout life unless it is lost by atresia or recruited for maturation. By contrast, the growing follicles have been activated to various stages of maturation in the process now generally called folliculogenesis. The “activated” pool includes primary, secondary, preantral, antral, and preovulatory or Graafian follicles (Gougeon, 1986). Several other terms have been proposed, including “ovulatory potential” (Findlay et al., 2015) or “dynamic reserve” (Monniaux et al., 2014), for the growing follicle pool that supports normal folliculogenesis and can potentially be ovulated or induced to maturity (e.g., in assisted conception).

In this discussion, adopting the preponderant view in the field, OR is considered as the size of the NGF pool in a woman’s two ovaries at any given age. This is the most valid measure of female reproductive lifespan.

However, the assessment of human OR remains technically problematic. Especially because follicles are unevenly distributed in the ovarian cortex (van Wagenen and Simpson, 1965; Sforza et al., 2003), invasive and partially destructive methods, such as ovarian biopsy, do not yield a reliable estimate of the OR (Lambalk et al., 2004; Lass, 2004), and direct *in vivo* counting is currently not possible.

A number of non-invasive procedures, including determination of ovarian volume, antral follicle count (AFC), and certain serum markers, have been proposed singly and in combination to assess the OR for individual women (American College of Obstetricians and Gynecologists (ACOG), 2015), but none of these procedures has been shown to be directly related to the size of the OR (Findlay et al., 2015). It has been observed that these procedures are a measure of “ovarian response” rather than a measure of OR (Nelson, 2014).

The most reliable route to assess the OR is to remove ovaries and carry out histomorphometry-based follicle counts in serial tissue sections of the entire ovaries (Tilly, 2003). To date, using this method on tissues retrieved post-mortem or post-oophorectomy, there have been six studies that estimated the OR in females at various chronological ages. Two of these studies have evaluated the OR in the phase of its formation (Block, 1953; Forabosco and Sforza, 2007), and four have focused on OR dynamics from birth to menopause (Block, 1952; Richardson et al., 1987; Gougeon et al., 1994; Hansen et al., 2008). These studies have shown that the OR increases dramatically from 15 weeks of post-conception (wpc) until the 34th wpc, and thereafter remains constant, with an average of about 680,000 NGFs, until at least 2 years after birth (Block, 1953; Forabosco and Sforza, 2007; Hansen et al., 2008). As for the OR in postnatal life, before puberty quantitative data are scanty. There are no data between 2 and 7 years, and from 7 to 12 years the measures show considerable variability (Block, 1952; Hansen et al., 2008). The available data indicate a limited decrement from early postnatal numbers. An average of ∼460,000 of follicles remains around puberty (age 12–14; Block, 1952; Hansen et al., 2008). Thereafter, the OR will decline continuously until menopause initiates at <1000 NGFs (Block, 1952; Richardson et al., 1987; Gougeon et al., 1994; Hansen et al., 2008).

The changing dynamics of OR are the consequence of two opposing processes that involve complex genetic and environmental factors: the formation of new NGFs and the recruitment of NGFs from the OR for maturation or atresia (Kerr et al., 2013). During this scenario, newly formed NGFs are maintained for various lengths of time during the reproductive lifespan (Adhikari and Liu, 2009; Reddy et al., 2010).

In summary, the size of the human OR during life is not constant. After a first prenatal step in which the OR is established, the size of the OR is kept constant during an intermediate perinatal step and then progressively decreases to values that no longer support ovulation (Pelosi et al., 2015).

The life course of ovarian function once the OR is established thus represents an unusual case in which aging has two components. The usual stochastic decline of function and activity seen in all physiological systems certainly occurs—as seen in the progressive loss of quality of oocytes (Henderson and Edwards, 1968; Tarín et al., 1998); but the major force is the programmed monthly recruitment of oocytes that progressively depletes the OR. In other words, the decline in the size of the OR drives reproductive aging (i.e., toward menopause). Menopause ensues when regular recruitment decreases follicle numbers below a threshold. This process is at least partially genetically determined. Thus, although it has much sharper timing in a population of women than other age-related declines, the dynamics of the reserve and the timing of menopause can be changed by mutations or environmental factors that alter the size of the initial reserve or slow the rate of recruitment/atresia.

The effects of prenatal and postnatal environmental factors on OR have recently been updated (Richardson et al., 2014), in this review we consider the genomic factors that influence OR dynamics during prenatal and postnatal life.

Studies have found many genes affecting OR: no single dominant gene has been identified as responsible for the life course of OR. We first consider the genomics of formation of new NGFs with the establishment of the OR, and then turn to the genomics of the recruitment of NGFs from the OR for maturation and atresia. We reference available data on human instances of reduced OR, and especially primary ovarian insufficiency (POI); but the vast majority of studies have been performed on animal models. There are obvious differences between the evolutionarily distant rodent models and human—including the time scale, the mouse estrus cycle, and some differences in gene usage noted in the text below. Nevertheless, the available comparative developmental and genetic data are consistent with very similar overall formation and fate of the OR.

## Genomics of the Establishment of the Ovarian Reserve

In every mammalian species, the OR is formed during definitive ovarian histogenesis, the last phase of ovary organogenesis (Ottolenghi et al., 2004), by a coordinated series of processes collectively referred to as follicular endowment (Hirshfield, 1991). NGF endowment begins with the formation, commitment, migration, and colonization of the gonad by a founder population of primordial germ cells (PGCs). After the development of the bipotential gonad, and female sex determination (see She and Yang, 2014), definitive ovarian histogenesis occurs, and the size of the OR is consolidated during follicle formation, now called follicular assembly (Skinner, 2005; Pepling, 2012).

### Genes Involved in Formation, Migration, and Gonad Colonization of PGCs

The somatic compartment and PGCs develop in different microenvironments, correlated with their idiosyncratic identities. During gastrulation—3 wpc in humans—a small population of PGCs develops in the extra-embryonic compartment, specifically at the base of the allantois in the endoderm of the dorsal wall of the yolk sac (Ginsburg et al., 1990). Subsequently, PGCs start to migrate to their final destination, the gonad, a process that is completed ∼6–7 wpc (Fujimoto et al., 1977; Kurilo, 1981). It is during this migration that PGCs start to proliferate, and they continue to multiply during the colonization of the gonad (Baker, 1963).

In mouse, the process is relatively compressed in its shorter lifespan, with PGCs developing starting at E6.25, migrating at ∼E8.5, and completely installed in the genital ridges by ∼E10.5 (Saitou and Yamaji, 2012).

Studies in the mouse have revealed some of the most critical signals for PGC specification, migration and proliferation. PGC specification is dependent on BMP signaling from the extra-embryonic ectoderm (Pelosi et al., 2011). Indeed, in *Bmp4*^-/-^ embryos PGCs fail to complete migration, and *Bmp4*^+/-^ ovaries show a reduced or absent population of PGCs (Lawson et al., 1999). Furthermore, deletion of other members of this family, such as *Bmp2* and *Bmp8b*, and of downstream mediators of the BMP pathway like *Smad1* and *Smad5*, produced similar phenotypes (Ying et al., 2000; Chang and Matzuk, 2001; Tremblay et al., 2001; Ying and Zhao, 2001). Subsequently, *Blimp1* expression is required to maintain PGC identity and inhibit differentiation into the mesodermal cell lineage (Ohinata et al., 2005).

Germ cells express important survival factors very early during and following their specification. They include the pluripotency-related *Oct4*. *Oct4* is necessary for embryo development and the formation of the pluripotent inner cell mass (Nichols et al., 1998). Conditional deletion of *Oct4* in PGCs also resulted in early apoptosis and resultant lack of germ cells in the adult ovary (Kehler et al., 2004).

*Nanos* genes are also important for specification and migration of PGCs. In *Drosophila* ablation of maternally-derived *Nanos1* leads to failure of PGC migration (Kobayashi et al., 1996). By contrast, deletion of mouse *Nanos1* did not affect germ cell development (Haraguchi et al., 2003; and *Nanos2* is expressed only in male PGCs). However, ablation of *Nanos3* caused defects in mouse PGC migration and proliferation, leading to sterility in both males and females (Tsuda et al., 2003).

Coordinated expression of *Kit* receptor and its ligand *Kitl*, encoded at the white spotting (W) and steel (Sl) loci, respectively, is indispensable for the survival, migration, proliferation, and overall proper establishment of ovarian PGC for future follicle assembly (Thomas and Vanderhyden, 2006). *Kit* is expressed in mouse germ cells starting at E7.5 during their migration to the ovary, while its ligand *Kitl* is expressed in somatic cells along the migratory route (Besmer et al., 1993). Several mutant alleles at the W or Sl loci have been described, and the type of mutation determines the severity of the phenotype, ranging from reduction of the number of PGCs reaching the gonad to reduced developmental capability of the germ cells (Russell et al., 1948; Reith et al., 1990; Buehr et al., 1993; Bedell et al., 1995). The mutant *steel panda* at the Sl locus causes a pronounced decrease in the size of the OR at birth, along with arrest of the remaining follicles at the primordial stage (Huang et al., 1993). By contrast, the Wv mouse variant, caused by a point mutation in the conserved ATP binding domain, produces a phenotype reminiscent of POI in women (Smith et al., 2012); the size of the OR in these mice is severely compromised at birth and exhausted by 2 months, and the mice display elevated gonadotropin levels, reduced estrogen and progesterone levels, and decreased bone density (Smith et al., 2012).

Factors involved in germ cell proliferation are also critical for the formation and maintenance of the initial population of germ cells. *Pin1* affects cell cycle regulation, and its ablation in mice caused fertility defects due to prolongation of the cell cycle with resultant slower proliferation. The features included diminished numbers of gonocytes and oocytes during embryonic development (Atchison et al., 2003). Similarly, deletion of *Pog*, which is involved in germ cell proliferation, led to sterility, with a smaller pool of germ cells at birth and no follicles in the adult ovary (Agoulnik et al., 2002).

### Genes Involved in Definitive Ovarian Histogenesis

Once the ovary has been formed and germ cells are committed to the female fate, ovarian development moves to the last phase of organogenesis (Ottolenghi et al., 2004), the definitive ovarian histogenesis with establishment of the OR. This occurs with the process of follicle histogenesis or follicular assembly (Pepling, 2012), starting about postnatal day 0 (P0, birth) in mice and before 13 wpc in human in the inner portion of the ovary, near the rete ovarii (Kurilo, 1981; Gondos et al., 1986; Sforza et al., 2003; Maheshwar and Fowler, 2008).

The solid cell mass of the bipotential gonad is progressively cleaved into elongate formations of germinal and somatic cells called “ovigerous cords” (Byskov, 1986).

The population of oogonia, derived from PGCs, expands dramatically through a series of rapid mitotic divisions with incomplete cytokinesis. This results in syncytial nests of oogonia called “germ cell nests” or “germ cell cysts.” In the human fetal ovary all oogonia display similar mitotic activity (Bendsen et al., 2006) until about 10 wpc, and then the first oogonia enlarge and become primary oocytes that initiate meiotic prophase I. Preleptotene cells are found as early as 9–10 wpc (Kurilo, 1981; Gondos et al., 1986; Bendsen et al., 2006).

Initially, meiosis is strikingly asynchronous, with more and more germ cells initiating meiosis while some oogonia are still expressing stem cell markers and continuing to proliferate until at least 16 wpc (Baker, 1963; Skrzypczak et al., 1981; Kerr et al., 2008). After the preleptotene stage, the primary oocytes rapidly proceed through the subsequent stages of prophase of the first meiotic division—i.e., leptotene, zygotene, and pachytene, reaching diplotene and the “resting” or “arrested” stage at which meiosis is blocked with the nucleus filled with decondensed chromatin. This state is also called “dictyate.”

We next discuss genes and processes within the oocyte that act in assembly of the primordial follicle pool.

#### Genes Involved in Germ Cell Survival, Meiosis, and DNA Damage Repair

Study of an anti-apoptotic *Bcl-x* hypomorphic mouse model showed that PGC migrated to the genital ridge by E12.5 but were lost by apoptosis by E15.5, suggesting the importance of *Bcl-x* for germ cell survival (Rucker et al., 2000). However, deletion of *Bcl-x* in postnatal oocytes did not affect the size of the OR or growing follicles, so that its specific action is apparently limited to embryonic development (Riedlinger et al., 2002).

In addition to apoptosis, autophagy is important in regulating oocyte and follicle development. Ablation of either of two autophagic factors, *Atg7* and *Becn1*, led to comparable loss of follicles by P1 (Gawriluk et al., 2011).

Connexin 43, encoded by *Gja1*, is one of the proteins forming the gap junctions that permit passage of molecules between cells in the ovigerous cords. The phenotype of *Gja1*^-/-^ mice is germ cell-specific and affects both sexes (Juneja et al., 1999). The number of PGC is reduced in embryonic gonads starting from E11.5, suggesting an indispensable function of connexins in communication between cells during PGC development.

During meiosis, germ cells undergo DNA double-strand breaks for recombination that potentiates genetic diversity in progeny. Mutations in genes involved in creation and repair of double-strand breaks negatively impact fertility and lead to POI (Pelosi et al., 2015). For example, *Spo11* is required to induce double-strand breaks during meiosis, and *Spo1*^-/-^ female mice have a smaller OR, with germ cell defects evident by E15.5 (Romanienko and Camerini-Otero, 2000). Meiotic defects also result from deletion of two other meiosis-specific genes. Premature loss of mouse germ cells that was complete by P4 was seen in *Msh4*^-/-^ and by 2 months in *Msh5*^-/-^ ovaries (de Vries et al., 1999; Kneitz et al., 2000).

Among additional meiotic genes, *Dmc1* encodes a protein needed for DNA strand exchange, and its deletion in mice also caused a reduction in mouse follicle numbers and follicle attendant on failure of chromosome synapsis (Pittman et al., 1998). Interestingly, both *MSH5* and *DMC1* have also been found associated with POI in women (Mandon-Pépin et al., 2008). Defective mouse chromosome synapsis also ensued when *Rec8*, a component of the cohesin complex, was ablated, causing loss of follicles by P5 (Xu et al., 2005).

A vital factor in DNA damage checkpoint control during meiosis is the ataxia telangiectasia mutated (*Atm*) gene, which is activated following double-strand breaks. Deletion of *Atm* in mice led to loss of follicles by P11 and resulted in sterility (Barlow et al., 1998). And in humans, ataxia telangiectasia, the loss-of-function mutation of *ATM*, is associated with ovarian dysgenesis with defects in PGC development (Miller and Chatten, 1967). Failure of crossover focus formation and generation of non-exchange mouse chromosomes also occurred after ablation of *Lsh*, another factor involved in the maintenance of genomic stability. Again, this led to oocyte loss and lack of follicle formation (De La Fuente et al., 2006).

*Cdk2* is involved more generally in cell cycle progression. It has nevertheless been found that *Cdk2* is dispensable during mitosis, but is critical for the completion of prophase I of meiosis, and *Cdk2* mutation caused complete germ cell loss by P2 (Ortega et al., 2003). A similar sterile phenotype resulted from ablation in mice of *Cbep*, which is involved in the regulation of the synaptonemal complex. *Cbep*^-/-^ female mice were sterile and contained only a few oocytes arrested in pachytene (Tay and Richter, 2001).

Ablation of *Brca1*—involved in DNA damage detection and repair, as well as cell cycle arrest—is embryonic lethal in mice (Shen et al., 1998). However, *Brca1* heterozygous mutant mice survived, though with a reduced OR seen as early as P5; by contrast, *Brca2* heterozygous or homozygous ovaries were indistinguishable from wild-type (Titus et al., 2013). Function of DNA repair genes, including *Brca1*, was shown to decrease with age, while double-strand breaks accumulated in mouse and human oocytes. Women with *BRCA1* mutations were also reported to have lower OR, inferred from AMH levels (Titus et al., 2013), but others observed no change in fertility or OR (Kotsopoulos et al., 2008; Moslehi et al., 2010; Pal et al., 2010; Valentini et al., 2013; Michaelson-Cohen et al., 2014). Understanding the role of *BRCA1/2* in menopause or POI is complicated by the lack of standardization in various studies, which assessed fertility or OR with various methods. In addition, several reports had few participants and stratification was not always the same. Also, the association between *BRCA1/2* and fertility may depend on other factors, even though several studies have reported earlier menopause or low OR in *BRCA* carriers (Rzepka-Górska et al., 2006; Oktay et al., 2010; Finch et al., 2013; Lin et al., 2013; Wang et al., 2014a). As one specific possible source of discrepancies in study results, *BRCA1/2* effects may be smaller in younger women and become more clinically evident in those approaching perimenopause (an age cohort that was not included in several of the studies).

Mutations in the Fanconi anemia complex of DNA damage genes are also implicated in the control of OR. Deletion of *Fanca* in mice led to reduced fertility and sterility by 21 weeks of age with the total depletion of ovarian follicles (Cheng et al., 2000). A very similar phenotype was also found in *Fancc*^-/-^ mice (Whitney et al., 1996); and deletion of *Fancg* caused comparable infertility at 21 weeks of age with a dramatic reduction in the OR (Koomen et al., 2002).

Heat shock proteins have additional functions in meiosis completion and oocyte development. *Hsf1* is a maternal effect transcription factor affecting the expression of several heat shock proteins including *Hsp90α*, *Hsp25*, *Hsp70.1*, and *Hsp105* (Metchat et al., 2009). When it was deleted in mice, meiotic defects in oocytes ensued, characterized by delayed G2/M transition, partial block of germinal vesicle breakdown, and asymmetrical division. Furthermore, *Hsf1* was required for embryonic development, eliciting a protective response against oxidative stress in the oocyte (Bierkamp et al., 2010).

Finally, mutations in several other meiotic genes have also been identified as associated with POI in humans. Caburet et al. (2014) demonstrated mutations in *STAG3* (cohesin)—thought to be involved in sister chromatid association—in a large pedigree evincing POI (“POF8” in OMIM 615723), and mice ablated for the gene were sterile. Mutation in the X-linked gene, *POF1B*, encoding a protein that interacts with actin filaments, causes “POF2B” (OMIM 300604; Lacombe et al., 2006); the authors hypothesized that *POF1B* could also function in the pairing of meiotic chromosomes, and alteration in its function could lead to drastically fewer oocytes being created. Wang et al. (2014b) found another meiotic gene, *HFM1*, mutated in two sisters with POI, and in another case among 69 analyzed (“POF9” in OMIM 615724). Mutation in *SYCE1*, essential for formation of the synaptonemal complex, can account for autosomal recessive POI in another family (de Vries et al., 2014).

#### Genes Involved in Follicular Assembly

During the progression to the dictyate state, the ovigerous cords are carved into clusters of germinal and somatic cells that give rise to the NGFs (Zamboni et al., 1980). The nascent NGFs emerge when the intercellular bridges between primary oocytes in meiotic prophase I break down while single oocytes become closely surrounded by a squamous layer of pre-granulosa cells. Outside the pre-granulosa layer a basement membrane encompasses and demarcates each newly formed NGF.

**FIGURE 1:**
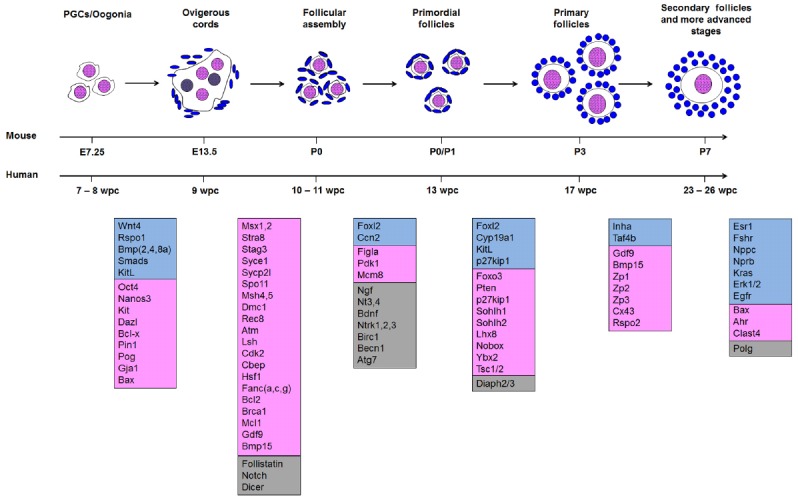
**Morphogenesis of follicles from the arrival of primordial germ cells (PGC) in the nascent ovary to secondary follicles.** Genes assigned to each transition are as specified in the text. Somatic genes are shown in blue; germ cell genes are shown in pink; and genes that are either expressed in both or whose site of expression has not been assigned are in gray. In the ovigerous cord, oocytes that are lost and do not form primordial follicles are colored gray.

Notably, assembly of a limited number of NGFs is accompanied by the degeneration of all the other oocytes in the clusters of cells surrounded by the squamous pre-granulosa cells and the basement membrane (Figure 1). More than 85% of germ cells degenerate, and it remains unclear whether there is specific selection for specific oocytes; whether the selection is random; whether those destroyed have any function in the process; or whether there may be insufficient auxiliary cells to support formation of more than a smaller number of follicles. Additionally, it is still unknown if the discarded oocytes provide any “nourishment” to surviving germ cells, as in *Drosophila* (reviewed in Pelosi et al., 2015). Nonetheless, several genes are known to be involved in the regulation of this process, and mutations in those genes have significant repercussions for the OR, as follows.

*Figla* is an oocyte-specific transcription factor required for primordial follicle formation (Soyal et al., 2000). When it is ablated in mice, oocytes fail to form follicles and eventually die soon after birth, leaving female but not male animals sterile. *Figla* is also important in maintaining femaleness and suppressing ectopic expression of male genes (Hu et al., 2010). It has been shown to directly regulate the expression of *Zp1*, *Zp2*, and *Zp3*, which encode the corresponding glycoproteins of the extracellular zona pellucida (Liang et al., 1997). Human *FIGLA* is also expressed in the oocytes of primordial follicles, suggesting a conserved function. Indeed, *FIGLA* mutation is a cause of POI in several Chinese POI patients (Zhao et al., 2008; “POF6” in OMIM 612310).

Also involved in primordial follicle formation, follistatin is involved in germ cyst breakdown and oocyte apoptosis. Mice expressing only a short isoform of follistatin displayed a larger OR, resulting from a reduction in germ cell apoptosis during germ cyst breakdown and a prolonged duration of the breakdown process itself (Kimura et al., 2011). However, these mice later lost the initial population of primordial follicles more rapidly than wild-type counterparts, so that other isoforms are likely required for the correct formation or maintenance of the OR.

The Notch signaling pathway is also implicated in the formation of the OR. Although mutations of various members of this family are embryonic lethal in mice (Conlon et al., 1995; Hrabĕ de Angelis et al., 1997; Jiang et al., 1998b; Xue et al., 1999; McCright et al., 2001), ovary cultures in which Notch signaling was disrupted by γ-secretase inhibitors contained fewer primordial follicles, with more of the oocytes persisting within the germ cysts (Trombly et al., 2009).

Neurotrophins, a family of growth factors involved in cell survival and development, are also activated in ovary development. Nerve growth factor (*Ngf*), its receptor (*Ntrk1*), and a related receptor (*Ntrk2*) have all been found to affect mouse primordial follicle formation. *Ngf*^-/-^ or *Ntrk1*^-/-^ ovaries showed a larger population of oocytes still inside germ cell cysts, and only a few primordial follicles were formed (Dissen et al., 2001; Kerr et al., 2009). In addition, neurotrophins *Nt4* and *Bdnf* are likely involved in follicular assembly and/or survival, because germ cell death was increased in cultures when signaling was blocked (Spears et al., 2003). Still another neurotrophin, *Nt3*, and its receptor *Ntrk3* also seem important for oocyte survival, and affect the transition of follicles from primordial to primary stage (Nilsson et al., 2009).

At the level of turnover of follicles, deletion of genes belonging to the apoptotic pathway also influenced the size and dynamics of the OR. *Casp2*^-/-^ mouse ovaries had more primordial follicles at P4, and germ cells were resistant to cell death following chemotherapy (Bergeron et al., 1998). Ovaries from mice in which the anti-apoptotic *Bcl2* was ablated showed a reduced number of primordial follicles, with little or no significant effect on growing follicles (Ratts et al., 1995). Roughly one third of primordial follicles were aberrant, with a single layer of cuboidal granulosa cells but without any oocyte within. However, morphometric analysis was done on adult ovaries, and no data on the initial size or the OR was provided. Thus it is unclear whether *Bcl2* is involved in germ cyst breakdown, oocyte survival during primordial follicle development, or both.

Deletion of the Bcl2-associated X protein (*Bax*), resulted in the formation of a larger mouse OR. *Bax*^-/-^ ovaries contained larger numbers of PGC during embryonic development, and had more primordial follicles than wild-type ovaries at P4 (Greenfeld et al., 2007). Although this contradicted a previous study (Perez et al., 1999; see below), it was inferred that the phenotype of increased follicular endowment was due to BAX regulation of PGC survival or proliferation during migration to the developing gonad (Greenfeld et al., 2007).

It should be noted that deletion of *Ahr*—a positive regulator of *Bax* (Matikainen et al., 2001)—produced mouse ovaries with no change during embryonic development (E18.5) but an increased population of primordial follicles at P2–P3 (Benedict et al., 2000). The number of primordial follicles returned to normal by P8 and remained very similar to wild-type until at least P32–P35. However, by P53 *Ahr*^-/-^ ovaries had fewer antral follicles, suggesting an action of AHR and BAX in follicle maturation as well as in primordial follicle endowment (Benedict et al., 2000).

Deletion of another apoptotic gene, *Mcl1*, caused a reduction in the OR that was significant as early as P7 and was almost total within 3 months. *Mcl1*^-/-^ oocytes displayed elevated mitochondrial dysfunction with elevated superoxide levels, and also showed chromosome abnormalities, all of which could plausibly activate the autophagic pathway (Omari et al., 2015).

A very different class of factors affecting formation of the OR comprises retrotransposable elements and their expressed genes. Overexpression of L1 led to significant increase in oocyte loss during NGF endowment, thus affecting the size of the OR (Malki et al., 2014). Interestingly, oocyte survival was recovered by treatment with the reverse transcriptase inhibitor AZT, demonstrating the specific action of L1 retrotransposons (Malki et al., 2014). The involvement of retrotransposons in germ cells and follicle dynamics could be more prevalent than generally thought. In human oocytes, there is some evidence of retrotransposon expression (Georgiou et al., 2009), and more studies are warranted, especially given the potential relevance for development and disease.

## Genomics of Folliculogenesis

In the overall process, once they are formed, NGFs can remain quiescent in mice for up to ∼18 months and in women for decades—or not even be used at all during reproductive life. They may undergo atresia before recruitment, or may instead be selected for maturation that will lead to the ovulation of one dominant oocyte competent for fertilization at each menstrual cycle. But the majority of assembled NGFs simply survive for long periods of time. If NGF dormancy is lost or they are damaged, follicular endowment is depleted by activation or death, respectively. This results in premature female reproductive aging and POI. One can therefore envisage two types of interventions that help to maintain the OR: (a) the maintenance of NGF quiescence and survival, and (b) the suppression of NGF activation (Adhikari and Liu, 2009; Reddy et al., 2010). Operationally the distinction is difficult, but stages in folliculogenesis can be discriminated, with many steps between the NGFs and the preovulatory or Graafian follicles, and those in turn can either ovulate their constituent eggs for fertilization or be lost by atresia.

Two events dominate folliculogenesis both in mouse and in human (Monniaux et al., 1997; McGee and Hsueh, 2000). In an initial step, NGFs are continuously recruited from OR (continuous recruitment) for their subsequent development until they have entered the antral cavity. In a successive step a few of the antral follicles are cyclically rescued to reach the preovulatory stage (cyclic recruitment).

In continuous recruitment the transition from primordial to primary follicles, termed follicle activation, results in morphological and physiological changes in both the germ and somatic cells. The volume of oocytes increases greatly, and the flattened pregranulosa cells surrounding the oocytes differentiate and proliferate into cuboidal granulosa cells. After activation follicles grow slowly (basal follicular growth), their growth rate tightly related to proliferation of granulosa cells. Most of the activated follicles undergo atresia.

In cyclic recruitment, in contrast, follicular growth is rapid and occurs by enlargement of the antrum (terminal follicular growth). In women, as in all mono-ovulatory species, only one of these follicles, the “dominant follicle,” is usually selected for ovulation of a fully competent oocyte every cycle, while the subordinate selected antral follicles are discarded by atresia.

Continuous recruitment with basal follicular growth is mainly under the control of growth factors of paracrine origin, and is gonadotropin-independent—though follicle-stimulating hormone (FSH) may exert an indirect mitogenic effect on granulosa cells by enhancing expression of growth factors or growth factor receptors. In contrast, cyclic recruitment with terminal follicular growth is strictly gonadotropin-dependent. FSH plays determinant roles in enhancing granulosa cell differentiation and survival. These actions are again mediated or modulated in an important way by paracrine factors, particularly steroids and growth factors. Luteinizing hormone (LH) stimulates steroidogenesis in theca cells and sustains terminal maturation of granulosa cells in pre-ovulatory follicles.

In the human ovary continuous recruitment begins early in prenatal life. Instead, cyclic recruitment occurs only after puberty. Primary follicles have been observed in 17-week old XX fetuses (Kurilo, 1981). By 23–26 wpc pre-antral follicles are also present, and after 35 weeks all follicle stages, primary to antral, can be observed (van Wagenen and Simpson, 1965; Sforza et al., 1993).

### Genes Involved in the Gonadotropin-Independent Phase

Based on both human studies and mouse models, factors involved in the transition from primordial to primary follicles include SOHLH1/2, NOBOX, LHX8, KIT, YBX2, and FOXO3—all expressed in the oocytes—and KITL and FOXL2, expressed in the granulosa cells. Signaling from other cell lineages may also influence NGF quiescence or recruitment, but specific actions are less studied.

One of the most powerful factors is Foxl2, which acts critically in several processes, including sex determination, ovary development and maintenance of gonadal femaleness. In humans, mutation of FOXL2 causes Blepharophimosis/Ptosis/Epicanthus Inversus syndrome (BPES), characterized by eyelid anomalies and POI (Crisponi et al., 2001). Foxl2 is never expressed in the testis, and is turned on specifically in granulosa cells of the ovary at the time of sex determination. When Foxl2 is ablated in female mice, granulosa cells in the ovary undergo partial sex reversal, characterized by expression of male specific markers including testis-determining Sox9. Joint ablation of Wnt4 and Foxl2 drives sex determination more decisively toward the testis fate during embryonic life, causing sex reversal that now extends to germ cells (Ottolenghi et al., 2007). Foxl2 also acts negatively on expression of Cyp26b1 and Sf1, which are required for male sex determination (Kashimada et al., 2011a; Takasawa et al., 2014). Furthermore, FOXL2 cooperates with BMP2 in regulating the correct expression of follistatin, which in itself is also required for female sex determination and ovary development (Kashimada et al., 2011b). *Foxl2* ablation causes primordial follicle failure in progression to the primary stage, resulting in female sterility, while the steroidogenic profile resembles that of testis, with the up-regulation of *Amh* and *Cyp11a1* and the inhibition of *Cyp19a1* (Uda et al., 2004; Ottolenghi et al., 2007). *Foxl2* expression remains obligatory throughout the entire reproductive lifespan (Garcia-Ortiz et al., 2009). Indeed, when *Foxl2* is ablated in the adult ovary the somatic compartment comprising granulosa and theca cells transdifferentiate to Sertoli-like and Leydig-like cells (Uhlenhaut et al., 2009).

*Sohlh1* and *Sohlh2* share a similar function in germ cells, and their ablation in mice results in the impairment of the transition from primordial to primary follicles, with subsequent premature follicle loss (Pangas et al., 2006; Choi et al., 2008a). *Sohlh1* has been shown to directly regulate *Lhx8* (Pangas et al., 2006), a transcription factor involved in oocyte survival; its deletion in mice also causes rapid oocyte loss (Choi et al., 2008b). When *Lhx8* is ablated, primordial follicles fail to progress to the primary stage and soon degenerate, leaving the ovary empty by P7 (Choi et al., 2008b). *Lhx8* directly affects expression of the homeobox gene *Nobox* (Park et al., 2012), another transcription factor that seems required for the transition to primary follicles. *Nobox*^-/-^ mouse ovaries appear very similar to their wild-type counterparts. However, the oocytes are developmentally impaired, with many germ cell cysts still present at P3 in *Nobox*^-/-^ ovaries; progression beyond the primordial stage is blocked, and follicles are nearly completely lost by P14 (Rajkovic et al., 2004).

Because these genes—*Sohlh1* and *Sohlh2*, *Lhx8*, and *Nobox*—belong to the same regulatory pathway, the similarities in phenotype when they are ablated are not surprising. Several of them have also been associated with POI in women. *LHX8* was not found changed in any of a cohort of Caucasian patients (Qin et al., 2008); and *SOHLH1* mutations or variants have not been evaluated, so that there may be a failure of ascertainment in that case. By contrast, eleven variants in the oocyte gene *SOHLH2*, including four mutations detrimental to its function, have been found associated with POI (Qin et al., 2014). As for *NOBOX* (“POF5” in OMIM 611548), it was first shown to be mutated in 1 of 96 POI cases analyzed (Qin et al., 2007). Strikingly, sequencing of *NOBOX* in a cohort of 178 women with POI found mutations in 12 (6.2%), and lesions in *NOBOX* may thus be a substantive cause of POI at a population level. All the characterized mutations compromised binding to the *GDF9* promoter (Bouilly et al., 2011), which would indeed arrest development. Other studies have reported deletions of *NOBOX* associated with POI (Rossi et al., 2008; Sehested et al., 2010).

*Ybx2*, also known as *Msy2*, is a germ cell-specific factor encoding a DNA/RNA binding protein. When mutated, it causes infertility in both male and female mice, likely due to instability of cytoplasmic transcripts (Yu et al., 2001). Ablation of *Ybx2* in the ovary results in follicle loss starting at around 3 weeks, accompanied by anovulation and absence of cumulus cells (Yang et al., 2005).

*Foxo3* is a potent transcription factor involved in follicle activation. In the mouse ovary, *Foxo3* is expressed exclusively in the oocytes of primordial and early growing follicles. FOXO3 is regulated by phosphorylation of AKT, which induces its translocation to the cytoplasm and therefore effectively blocks its nuclear function (John et al., 2008). When *Foxo3* is ablated in mice, all the primordial follicles start growing uncontrollably and the ovary becomes void of follicles by 15 weeks (Castrillon et al., 2003). It has been shown that *Galt1* is a downstream target of *Foxo3*. Prolactin signaling through the short form of its receptor causes *Foxo3* down-regulation, and consequently *Galt1* repression, again leading to accelerated follicle recruitment and POI (Halperin et al., 2008). This is of particular interest because human *GALT* deficiency causes galactosemia, another cause of POI (Pelosi et al., 2015; OMIM 230400).

Because of its function in controlling the OR dynamics, lesions in the human *FOXO3* seem a plausible candidate to cause POI. However, studies in women have been inconclusive (Gallardo et al., 2008), consistent with the finding that *FOXO3* does not seem to be expressed in human oocytes (Tarnawa et al., 2013). In fact, *FOXO3* has been found reported to be expressed in granulosa cells rather than oocytes in human ovaries, where instead *FOXO1* may take over its function (Tarnawa et al., 2013). Conversely, *Foxo1* is expressed in mouse somatic cells but not oocytes, suggesting a differentially evolved mode of regulation between human and mouse ovaries. No involvement of *Foxo1* in POI has been reported, but this may rather reflect the widespread requirement for FOXO1 action in many tissues.

One might anticipate that other factors belonging to the PI3K/AKT pathway might participate in controlling size of the OR. Indeed, one of these factors, *Pten*, encodes a lipid phosphatase that negatively regulates PI3K, and is also involved in setting the rate of follicle activation in mice: oocyte-specific deletion of *Pten* again leads to massive primordial follicle recruitment (Reddy et al., 2008). However, as with *FOXO* genes, changes in *PTEN* have at least as yet not been found in human POI (Shimizu et al., 2009; Zhao et al., 2011).

Quiescence of primordial follicles is further regulated by a complex of TSC1 and TSC2, which act by inhibiting the activity of mTOR complex1. Deletion of *Tsc2* in mouse oocytes caused increased mTOR activity followed by accelerated recruitment of the OR starting by P23 and follicle depletion by 4 months (Adhikari et al., 2009). A similar POI-like phenotype was also observed in mice with oocyte-specific deletion of *Tsc1* (Adhikari et al., 2010). Interestingly, double knock-out of *Tsc1* and *Pten* enhanced the effect on follicle recruitment compared to single gene mutants (Adhikari et al., 2010). These results suggest an additive effect between *Tsc1* and *Pten*, consistent with overlapping functions of the mTOR and AKT pathways in regulating the dynamics of the OR. Notably, another factor involved in both mTOR and AKT signaling is PDK1, and oocyte-specific deletion of *Pdk1* again caused accelerated mouse follicle loss, consistent with a role for this gene as well in follicle survival (Reddy et al., 2009). Moreover, the authors suggested a model in which PDK1 activity lies at the decision point between loss and activation of the OR.

P27KIP1 also participates in maintaining NGFs in a quiescent state. In *p27kip1*^-/-^ mice, primordial follicles were prematurely recruited and subsequently lost, causing POI comparable to that seen in *Foxo3*^-/-^ ovaries (Rajareddy et al., 2007). However, it was shown that P27KIP1 and FOXO3 acted independently. In addition, unlike *Foxo3*, *p27kip1* is expressed in both oocytes and granulosa cells of primordial and growing follicles, thereby affecting proliferation and survival of granulosa cells as well (Rajareddy et al., 2007).

Hippo signaling provides still another pathway inhibiting follicle recruitment (Kawamura et al., 2013). This pathway is characterized by the action of MST1 and MST2—orthologous to *Hpo* (Hippo) in *Drosophila*—which along with SAV1, phosphorylate LATS1/2. That leads to further phosphorylation and later degradation of YAP and TAZ, and results in suppression—or at least limitation—of follicle recruitment. When Hippo signaling is disrupted in mice, YAP and TAZ are active and interact with TEAD transcription factors, activating transcription of downstream target genes including *Ctgf* and *Naip* (Kawamura et al., 2013). CTGF is involved in follicle development, and *Ctgf*^-/-^ mice were subfertile, had decreased ovulation rates, and increased apoptosis of granulosa cells (Nagashima et al., 2011). Furthermore, statistically significant copy number variations of *NAIP*, which is involved in inhibition of apoptosis, were found in POI patients of Caucasian origin (Aboura et al., 2009).

Two genes acting upstream in the Hippo pathway, *DIAPH2* and *DIAPH3*, were also found associated with menopause. Castrillon and Wasserman (1994) had identified the *dia* locus in *Drosophila* by screening for male sterility mutations, and found alterations in cytokinesis associated with mutated *dia* alleles. In women, the X-linked gene *DIAPH2* was disrupted by a breakpoint in a family with POI (“POF2A,” OMIM 300511; Bione et al., 1998), and more recently variants in the gene were also found associated with the regulation of the age of menopause (He et al., 2010). Variants of *DIAPH3* have now been reported associated with both timing of menopause and size of the OR (Schuh-Huerta et al., 2012).

*Amh*—another member of the TGFβ superfamily—starts to be produced in granulosa cells of mouse follicles starting at the primary stage (Durlinger et al., 2002a). In *Amh*^-/-^ ovaries, the number of growing follicles was larger than control, whereas depletion of primordial follicles became significant by 4 months of age (Durlinger et al., 1999). When neonatal ovaries were cultured *in vitro* in presence of AMH, growing follicles were reduced by 40–50% after 2 and 4 days (Durlinger et al., 2002b). In addition, when preantral follicles were cultured in presence of AMH, FSH-induced follicle growth was inhibited. A similar result was obtained when *Amh*^-/-^ mice were treated with FSH: follicle recruitment was greater than in wild-type females (Durlinger et al., 2001). These results confirm a dual role for AMH: during gonadotropin-independent recruitment it inhibits the transition from the primordial to the primary stage, whereas in the gona-dotropin-dependent phase it antagonizes responsiveness to FSH.

Follicle recruitment as well as folliculogenesis are dominated by communication between oocyte and granulosa cells.

Two important oocyte factors involved in follicle growth are *Gdf9* and *Bmp15*, both members of the transforming growth factor beta superfamily. They are 52% homologous and have similar functions, primarily involved in the progression of follicle maturation (Yan et al., 2001).

*Gdf9* is necessary for fertility, because its ablation in mice causes a block in follicle growth at the primary stage (Yan et al., 2001). Additionally, *Gdf9* actively induces granulosa cell proliferation; formation of the theca layers and promotion of androgen biosynthesis from theca cells (Solovyeva et al., 2000); and expansion of the cumulus cells. Hence it is actively involved in the communication between the oocyte and surrounding somatic cells (Dong et al., 1996; Elvin et al., 1999).

By contrast, *Bmp15* ablation in mouse does not result in infertility. However, mice are subfertile, have a reduced litter size, and display impairment of ovulation and fertilization, suggesting a prevalent role of *Bmp15* in the peri-ovulatory period (Yan et al., 2001).

Interestingly, *Gdf9* and *Bmp15* both appear to be involved in the breakdown of ovigerous cords as well, because mouse mutant ovaries showed significant numbers of follicles containing multiple oocytes (Yan et al., 2001).

Mutations in *GDF9* have also been associated with POI in several populations, including missense mutations in a cohort of Indian patients (Dixit et al., 2005) as well as POI cases of Caucasian, Chinese, and African origin (Laissue et al., 2006; Kovanci et al., 2007; Zhao et al., 2007). *BMP15*, an X-linked gene, has also been associated with a substantial number of instances of POI (“POF4” in OMIM 300510). Rossetti et al. (2009) screened 300 unrelated idiopathic overtly POI women and identified six non-synonymous *BMP15* variants in 29 of them. Other reports have noted additional associated mutations in *BMP15* (Di Pasquale et al., 2004; Dixit et al., 2006; Laissue et al., 2006).

A member of the connexin family, connexin 43, is directly involved in cell-cell communication and is necessary for the correct development of growing follicles. Although ovaries from *Cx43*^-/-^ mice seemed grossly similar to wild-type up to several weeks after birth, growing follicles failed to complete maturation to the Graafian stage. In addition, mutant ovaries showed premature luteinization, and oocytes were likely impaired in completing meiosis to achieve competence.

Related to the interplay between oocyte and somatic cells, growth factor *Rspo2* is also involved in folliculogenesis, and particularly in inducing the development from primary follicles to more advanced developmental stages (Cheng et al., 2013). RSPO2 is produced by the oocyte from the primary stage onward, and functions both *in vitro* and *in vivo* as a paracrine factor able to stimulate follicle maturation. Additionally, when human cortical cubes where transplanted into the kidney capsule of immune-deficient mice, RSPO2 treatment was effective in promoting the transition of human primary to secondary follicles (Cheng et al., 2013). Interestingly, a mouse model hypomorphic for *Rspo2* showed that in addition to skeletal malformations, females had a reduced reproductive lifespan resembling POI (Bell et al., 2003). However, *RSPO2* function in humans still remains to be assessed, and thus any association with POI is not known.

### Genes Involved in the Gonadotropin-Dependent Maturation Phase

Maturation occurs with the integration of extra-ovarian signals and intra-follicular factors to determine whether an antral follicle will continue to develop or be diverted into atretic pathways. Cyclic follicular recruitment is characterized by induction of expression of mRNAs encoding a range of steroidogenic enzymes, gonadotropin receptors, and local regulatory factors. FSH provides the primary extra-ovarian driver for cyclic follicular recruitment, and LH (lutropin) is required for further development of follicles to the pre-ovulatory stage. FSH acts through membrane-associated granulosa cell receptors (FSHR) to stimulate granulosa cell proliferation and differentiation. The most responsive follicle at the beginning of the cycle is the first to produce estrogen and express LH receptor (LHR) on granulosa cells.

Follicle-stimulating hormone production is itself regulated by inhibin α (*Inha*), a glycoprotein member of the TGFβ superfamily (Vale et al., 1986). Inhibins are considered among the biomarkers of OR in predicting human reproductive lifespan (Steiner, 2013). *Inha*^-/-^ mice are infertile due to compromised follicular development and the formation of mixed tumors of granulosa and theca or undifferentiated stromal cells (Matzuk et al., 1992). After screening patients with POI or primary or secondary amenorrhea, Dixit et al. (2004) found a missense mutation in *INHA* associated with POI, though no associated variants were found in *INHBA* or *INHBB*. Confirming the positive finding, other reports have also found mutations and variation of *INHA* involved in POI in several populations (Shelling et al., 2000; Marozzi et al., 2002; Harris et al., 2005; Woad et al., 2009).

Disruption of the inhibin-activin-follistatin pathway also occurs in *Taf4b*^-/-^ female mice (Freiman et al., 2001). TAF4B is a subunit of transcription factor TFIID, which participates with RNA polymerase II, and was found specifically expressed in ovarian granulosa cells. *Taf4b*^-/-^ mice were sterile, and mutant ovaries showed a sharp decrease in numbers of growing follicles and failure of oocyte maturation and/or fertilization. No follow-up has reported whether TAF4B affects the transcription of a targeted subset of genes.

Follicle-stimulating hormone is also a predictor of reproductive lifespan, controlling development of follicles to the antral stage, as shown by targeted mutation of *Fshb* in mice (Kumar et al., 1997). Furthermore, the same phenotype was observed by disruption of the FSH receptor, confirming the importance of this signaling pathway for the completion of folliculogenesis (Dierich et al., 1998). Missense mutation in *FSHR* associated with POI has been reported in Finnish patients (Aittomaki et al., 1995; Jiang et al., 1998a; Doherty et al., 2002). However, some *FSHR* variants have been found in both POI cases and controls (Sundblad et al., 2004), and mutations causative of POI are rare (Layman et al., 1993; da Fonte Kohek et al., 1998). Variants in *FSHB* were nevertheless found associated with age of menopause in Caucasian women (He et al., 2010), and one of those single nucleotide polymorphisms was reported associated with age of menopause in women of African origin as well (Spencer et al., 2013).

FSH increases the expression in mouse granulosa cells of C-type natriuretic peptide (CNP)—encoded by the *Nppc* gene—but not its receptor NPRB (Sato et al., 2012). Unlike FSH signaling, which is mediated mainly through cAMP, CNP uses cGMP specifically as second messenger (Sato et al., 2012). In *Nppc* and *Nprb* hypomorphic mice, meiotic arrest was reversed prematurely, and although ovulation occurred seemingly normally, female mice were unable to deliver offspring. These results suggested the importance of CNP/NPRB signaling for correct and synchronized maturation of oocytes and cumulus cell differentiation from granulosa cells (Zhang et al., 2010; Kiyosu et al., 2012). In addition to skeletal defects and survival problems, *Nprb*^-/-^ mice also showed arrest of ovarian follicles at the pre-antral stage (Tamura et al., 2004).

The LH signaling pathway is responsible for inducing ovulation and luteinization through the coordinated cooperation of theca and granulosa cells. LH stimulates theca cells to produce androgens, which are then converted into estradiol by the granulosa cells (Pelosi et al., 2015). Consequently *Lh*^-/-^ mice are infertile, and mutant females show degenerating antral follicles and an absence of corpora lutea (Ma et al., 2004). In addition, levels of estrogen and progesterone were lower than in wild-type mice. Confirming the hormonal basis of the phenotypes, *Lh*^-/-^ mice can be rescued by chorionic gonadotropin administration. *Lhr*^-/-^ mice are comparably infertile, and ovaries contained follicles only up to the early antral stage, with no corpora lutea and elevated LH (Zhang et al., 2001). In humans, a variant has been found associated with the timing of menopause (He et al., 2010).

In granulosa cells, the surge of LH ultimately leads to the activation of downstream factors including RAS, ERK1/2, and EGFR. When a constitutively active form of *Kras* was expressed in granulosa cells, transgenic mice showed impairment of ovulation, decreased ERK1/2 activity, and elevation of Protein kinase B, PKB (AKT) function. The result in the mice was subfertility and POI (Fan et al., 2008). Disruption of *Erk1/2* in granulosa cells itself caused female infertility due to ovulation failure; and it was further shown that *Erk1* and *Erk2* are required for the LH-induced resumption of meiosis in oocytes and the attendant ovulation and luteinization (Fan et al., 2009).

C/EBPβ, a downstream mediator of *Erk1/2*, is also important in the response to the LH surge. Indeed, deletion of *Cebpb* in mouse granulosa cells caused subfertility, whereas *Cebpa/b* double knock-outs were sterile. *Cebpa/b*^-/-^ ovaries failed to ovulate and were devoid of corpora lutea, indicating the importance of *Cebpa* and *Cebpb* in regulating the terminal differentiation of granulosa cells during the luteinization process (Fan et al., 2011). Targeted disruption of *Egfr* in granulosa cells caused impaired oocyte meiotic resumption and impaired cumulus expansion, with reduced ovulation and fertility (Hsieh et al., 2011); the LH-induced activation of ERK1/2, p38MAPK, and connexin-43 was also impaired.

Estrogen signaling is also critical for folliculogenesis and reproduction. Disruption of the estrogen receptor gene in mice caused infertility; arrest of folliculogenesis with absence of corpora lutea; and absence of mating behavior that was not restored even with estrogen supplementation (Lubahn et al., 1993). Additionally, deletion of *Esr1?* in theca cells resulted in premature loss of mouse fertility similar to POI (Lee et al., 2009). In fact, polymorphisms of *ESR1* in women have been found associated with POI and idiopathic infertility (Bretherick et al., 2008; Yoon et al., 2010; Du et al., 2011; Cordts et al., 2012; M’Rabet et al., 2012).

### Other Genes Involved in Folliculogenesis

Variants in still other genes have been found causative of POI, but their involvement in ovarian development has not yet been studied extensively. Duncan et al. (2012) reviewed 13 cases of mutations in *POLG*, the mitochondrial DNA polymerase, that were associated with POI—and frequently also with neurological symptoms and male infertility. Current data indicate that *POLG* variants can also affect the timing of normal menopause (Stolk et al., 2012; Shen et al., 2013). One study found no association (Bonomi et al., 2012), but examined only a small number of cases. As another example, *Clast4* is highly expressed in mouse oocytes during follicle maturation, but its function is still poorly understood (Villaescusa et al., 2006). However, dominantly inherited mutation of its human ortholog—protein initiation factor 4E nuclear import factor 1 (EIF4ENIF1)—was inferred as causative of POI in a 3-generation family (Kasippilai et al., 2013).

Although endocrine disorders, including hypothyroidism, adrenal insufficiency, and hypoparathyroidism, all affect the hormonal profile required for fertility, only a few POI cases have been associated with mutations in genes of the endocrine system. Most suggestively, *NR5A1* (Steroidogenic factor Sf1), a gene involved in gonadal differentiation and steroidogenesis (Tremblay and Viger, 2003; Takasawa et al., 2014), is mutated in “POF7” in OMIM 612964. Lourenco et al. (2009) identified missense, frameshift, and in-frame mutations in *NR5A1* in families with anomalies of ovarian development and function, and in 2 of 25 sporadic cases of POI.

As for other endocrinological genes, Bhangoo et al. (2007) reviewed cases of females with deficiency in the steroidogenic acute regulatory (*STAR*) protein who showed recessive steroidal hormone and adrenal insufficiency leading to POI. Zangen et al. (2011) attributed POI in a case of Arab Palestinian origin to mutation in *PSMC3IP*, abolishing co-activation of estrogen-driven transcription. In another study, no mutations of *PSMC3IP* were found in Swedish POI patients (Norling et al., 2014), but ascertainment in such studies is often limited by small numbers of patients and the large number of known “POI” genes.

## Non-Coding RNAs

As in other physiological systems, it is increasingly evident that small non-coding RNAs are essential in the regulation of ovarian physiology. The classes expressed in the ovary include piwi interacting RNAs (piRNAs); small interfering RNAs (siRNAs); microRNAs (miRNAs); and long non-coding RNAs. We cannot comment on analyses of lncRNAs in the dynamics of the OR, because systematic studies have not yet been reported. It is currently believed that piRNAs are required primarily for mammalian male spermatogenesis rather than oocyte or ovary development. Several mouse models showed important roles of piRNAs in the male germline (Deng and Lin, 2002; Kuramochi-Miyagawa et al., 2004; Aravin et al., 2007; Carmell et al., 2007). Instead, despite known functions in oogenesis in lower organisms (Thomson and Lin, 2009), a study in female mice showed that deletion of a piRNA interacting protein had no effect on female germline development (Kuramochi-Miyagawa et al., 2004). Consistent with these findings, *Dicer 1* (see below) affects ovarian function, but is required for siRNAs and miRNAs, and not for piRNA formation. Thus, the presence of piRNAs in the ovary may be adventitious.

A variety of other mouse models have explored the involvement of miRNAs in diverse cells and processes in the ovary.

Conditional deletion of *Dicer1* in oocytes resulted in sterility (Murchison et al., 2007). Although ovaries were grossly normal and responsive to gonadotropins, oocytes were arrested in meiosis I and showed spindle and chromosome congression defects. Mutant oocytes had increased numbers of maternal transcripts and up-regulation of genes involved in microtubule organization, suggesting that regulated degradation of these classes of mRNAs promoted by siRNAs, and miRNAs may be critical (Murchison et al., 2007).

Conditional deletion of *Dicer1* in granulosa cells also resulted in sterility (Nagaraja et al., 2008). In addition to defects in the uterus and oviduct, granulosa cells in the ovary showed increased apoptosis, and fewer oocytes ovulated following superovulation, with some oocytes remaining trapped inside luteinized follicles (Nagaraja et al., 2008). Examination of a different group of ovaries in which *Dicer 1* was ablated only in granulosa cells revealed that primordial follicle endowment was increased in *Dicer1*^-/-^, leading to accelerated follicle recruitment that was, however, negated by an increase in follicle degeneration (Lei et al., 2010).

The generation of a mouse model with a hypomorphic *Dicer1* allele provided further information about the role of small RNAs in the ovary. Mutant males were fertile, whereas females were sterile, and this was specifically due to DICER1 deficiency, because mutant females that received ovary transplantation from a wild-type mouse were able to deliver normal offspring (Otsuka et al., 2008). The mutant females showed luteal insufficiency due to impaired angiogenesis of the corpora lutea. The authors attributed this phenotype to the lack of two miRNAs, miR17-5p and let7b, that regulate antiangiogenic factor Timp1. However, injection of these miRNAs failed to completely rescue pregnancy in mutant females, so that other small RNAs may be involved.

The *lin28*/*let7* system specifically affects for mouse germ cell development and OR formation. Male and female mice ablated of *lin28*, a RNA-binding protein that represses *let7*, showed early defects in PGC proliferation. Whereas in males this was partially corrected in adult life due to the continuous proliferation of spermatogonial stem cells, females—whose germ cells do not proliferate in the adult—formed a smaller OR and had reduced fertility (Shinoda et al., 2013). This phenotype was replicated by overexpression of *let7*, confirming the specific action of the *lin28*/*let7* system in regulating the size for the OR (Shinoda et al., 2013).

As a clue to timing, hormones were found to regulate miRNA expression in the granulosa cells of the mouse ovary. After *in vitro* FSH treatment 31 miRNA species were differentially expressed in granulosa cells. Interestingly, the expression of miR-29a and miR-30d decreased significantly 12 h after FSH supplementation, and then increased again by 48 h, suggestive of a complex feedback circuit (Yao et al., 2010b). Following treatment with LH/hCG, Fiedler et al. (2008) found that 13 miRNA’s were differentially expressed in peri-ovulatory granulosa cells, and in particular miR132 and miR-212 showed higher expression. The authors found 77 putative mRNA targets of miR132 and miR-212, with CTBP1, a known miR-132 target, among them.

More recently, *in vitro* experiments on primary human granulosa cells that were transfected with anti-miR-15a, or alternatively with pre-miR15a, showed that this miRNA affects a series of processes including proliferation, apoptosis, and hormone synthesis (Sirotkin et al., 2014). The same investigators had previously identified a group of microRNAs involved in the repression of estrogens, androgens, and progesterone (Sirotkin et al., 2010). MiR-21 is also involved in the apoptosis of granulosa cells during luteinization, but no target of its action has thus far been identified (Carletti et al., 2010).

*In vitro* experiments in which miR-378 was over-expressed or inhibited in porcine granulosa cells revealed a role in regulating aromatase—and thus estradiol—levels. In addition, two miR-378 binding sites that were critical for its function were identified in the aromatase coding region (Xu et al., 2011). It was also shown that miR-133b binds to *Foxl2* mRNA, thus inhibiting its expression and its ability to induce expression of StAR and aromatase (Dai et al., 2013). Another miRNA, miR-224 was shown to be induced by TGFβ, and subsequently increases estradiol production by granulosa cells *in vitro* through elevation of aromatase mRNA levels (Yao et al., 2010a).

Experiments in the bovine ovary provided further information about important miRNA action in the ovary and early embryo development. miR-196a is a regulator of NOBOX expression in the bovine ovary. A putative miRNA recognition element was identified in the 3′ UTR of NOBOX mRNA, and its binding activity was confirmed by luciferase assays. Injection of miR-196a in bovine embryos significantly decreased NOBOX expression at both the mRNA and protein level (Tripurani et al., 2011). Because *NOBOX* variants are associated with human POI, it would be particularly interesting to assess the function of miR-196a in POI patients.

In addition, bovine miR-181a was found to be involved in the regulation of oocyte-specific NMP2, a protein critical for DNA remodeling. MiR-181a is a maternally inherited miRNA that binds to the 3′ UTR of the NMP2 transcript and is involved in early embryo development (Lingenfelter et al., 2011). Ma et al. (2011) found a second role for miR-378, involvement in apoptosis during luteinization, and identified IFNGR1 as a putative miR-378 target. It remains to be seen if these functions are conserved in other species.

The majority of experiments thus far have been conducted *in vitro* on isolated cells, and most importantly, the function of these miRNAs has not been evaluated with regard to the OR. Nevertheless, these results demonstrate the relevance of small regulatory RNAs in important processes of ovarian function. Thus, their validation with *in vivo* models could provide exciting results, identifying additional points of control in the regulation of fertility and reproductive lifespan. One potential role of miRNA’s in OR dynamics might well be in the initial recruitment of primordial follicles. Consistent with such a role, when mouse P3 and P5 ovaries were compared, 24 miRNA’s were found differentially expressed. The authors showed that inhibition of miR-145 induced primordial follicle recruitment, and suggested that miR-145 as particularly associated with regulation of the TGFβ pathway in granulosa cells of primary follicles (Yang et al., 2013). Microarray analysis of RNA profiles in a rat model of POI identified 63 miRNA’s that were differentially expressed in POI vs control ovaries. Once again, apoptosis was one of the main putative pathways regulated by some of these miRNA’s, along with other pathways including hormone response and prostaglandin biosynthesis (Kuang et al., 2014).

Recently, some miRNAs have also been reported to be correlated with POI in human. Zhou et al. (2011) measured miRNA expression in the serum of Chinese normal and POI patients, and found 12 miRNA’s that were differentially expressed (Zhou et al., 2011). Another study with a similar approach found differentially expressed miRNA’s in plasma of POI patients, suggesting that several pathways, including AKT signaling and steroid hormone receptor pathways, might be regulated by these miRNA’s. Additionally, the authors provided evidence for miR-23a function in granulosa cell apoptosis and suggested a role in POI (Yang et al., 2012). Rah et al. (2013) investigated the association of miRNA variants in Korean POI patients and described risk and protective effects of combinations of three miRNA polymorphisms. However, none of the polymorphisms was independently associated with POI (Rah et al., 2013). Finally, a microarray analysis of POI patients identified a panel of 51 differentially expressed miRNA, several of which were further validated. In particular, miR-22-3p, involved in apoptosis and tumorigenesis, was suggested to be protective against POI and negatively correlated with serum FSH (Dang et al., 2015).

One caveat for many of the miRNA determinations during ovary aging is that they do not take into account the changing composition of the ovary as the OR declines in normal aging or in POI. This makes further work necessary to determine which variations in miRNA may be truly associated with POI from those that merely reflect the loss of follicles.

## Interventions Affecting the Time of Menopause in Mouse Models

Because women in modern societies are reproducing later (Martin et al., 2011), investigations of possible interventions to prolong reproductive lifespan are increasing. Understandably, such studies have been performed in animal models to try to understand the mechanisms involved. However, a complication is that in some mammals the ovary seems to be able to “sense” the size of the follicle reserve and to compensate for both innate and induced changes. The precise molecular pathways involved in this “sensing” mechanism, and why such a mechanism exists, are unknown. It may be necessary for the selection of the best quality oocytes or to limit premature decline in numbers of primordial follicles—that is, precisely in the mechanism whereby the time of menopause is so narrowly restricted.

During embryonic development, for example, XO female mice initially generate more germ cells than XX mice (Burgoyne and Baker, 1985). However, the atresia following the peak production of germ cells is much more extensive in the XO mice, leaving the perinatal XO ovary with half the number of primordial follicles of the XX ovary. Consequently, due to the reduced OR, XO female mice become sterile earlier than XX mice. However, the rate of depletion of the OR was found to be the same in XO vs XX mice (Burgoyne and Baker, 1981). In other words, the same constant proportion of primordial follicles is lost over time, allowing for a delimited period of fertility in XO animals.

Interventions aiming rather to increase the OR have been successful, but only temporarily. Animals transgenic for the anti-apoptotic *Bcl2* were born with a significantly larger population of follicles (Flaws et al., 2001). This was likely due to BCL2 protection against apoptosis during embryonic and perinatal development. However, a compensatory mechanism was again soon evident in this case, with the OR returning to wild-type levels by 30–60 days after birth (Flaws et al., 2001). Similar results were obtained when pups were administered activin A, a factor involved in germ cell proliferation before primordial follicle formation (Bristol-Gould et al., 2006). The larger numbers of follicles generated in treated mice did not persist until puberty but were rather lost, again consistent with the existence of a “census mechanism” that could aid in germ cell quality control.

The size of the primordial follicle pool of *Casp2*^-/-^ mice was also larger than wild-type in P4 ovaries, and oocytes were resistant to doxorubicin-induced apoptosis (Bergeron et al., 1998). However, the dynamics of the OR was not evaluated, and it was not shown if this phenotype persisted in older ovaries.

Another gene involved in cell death, *Smpd1*, encodes sphingomyelinase phosphodiesterase 1, an important factor in generating the signal for apoptosis. *Smpd1*^-/-^ ovaries showed a down-regulation of apoptosis at E13.5 *in vitro*, and *Smpd1*^-/-^ mice had an increased population of primordial, primary, and pre-antral follicles both at P4 and P42. These findings suggested a critical role for SMPD1 in controlling the size of the OR. However, follicle numbers in older ovaries were not assessed (Morita et al., 2000).

More lasting effects were demonstrated by ablation of pro-apoptotic *Bax* in mice (Perez et al., 1999). Although the number of follicles was the same in *Bax*^-/-^ and WT mice at birth, significant follicle numbers and maturation were still seen up to 640 days post birth. However, aged *Bax*^-/-^ female mice failed to become pregnant by natural reproduction, suggesting that quality or performance was defective.

Another model manipulated *Foxo3*. Generation of transgenic mice harboring a constitutively active form of *Foxo3* (engineered so that it could not be inactivated by phosphorylation) successfully increased the ovarian follicle reserve throughout mouse reproductive life (Pelosi et al., 2013). In addition, age-related increase of gonadotropin levels was diminished in transgenic animals and fertility was increased by up to 49%. Interestingly, increased fertility remained significant throughout the entire period of observation (Pelosi et al., 2013).

Ablation of interleukin 1 (*Il1*) also resulted in prolongation of fertility in mice. There was no difference in follicle numbers between *Il1*^-/-^ and wild-type ovaries throughout reproductive lifespan (apart from a transient increase in secondary and antral follicles at 2.5 months that was later compensated). However, the increase in fertility observed at 2.5 months of age persisted until 12 months. This suggested that the observed decreased expression of pro-inflammatory cytokines in *Il1*^-/-^ ovaries might affect follicle dynamics (Uri-Belapolsky et al., 2014).

Finally, inactivation of the mTOR pathway by rapamycin also preserved primordial follicles in young 20 week old rats (Zhang et al., 2013), and caloric restriction gave similar results (Li et al., 2015). Especially if these effects prove to be long-lasting, these approaches would provide epidemiological interventions that are potentially at hand.

## Conclusion

Appropriate to the critical process by which reproduction and continuity of a species are maintained, formation and stabilization of follicles is intricate. The ovary, like all organs, contains cells of numerous lineages that must organize and cooperate in coordinated regulation to produce competent and optimal quality germ cells for fertilization and development of embryos. A wide variety of pathways are coordinated, and the OR is thus sensitive to many insults, that are often dosage-dependent. Thus, in general, genes that cause POI in humans have loss-of-function in only one allele; the resultant heterozygous women have the pathognomonic reduction in OR; and as might be expected, the knockout phenotypes in corresponding mouse models are far more severe, frequently showing complete loss of OR.

Although many genes involved in ovary development and function have been characterized, many more are vital participants in the implicated pathways, and many other genes and pathways that affect individual lineages and their interactions are certainly still unidentified.

Among the important open questions is the extent to which epigenetic changes through life may affect folliculogenesis and the OR. Thus far, a few studies have examined DNA methylation and follicle dynamics following exposure to environmental toxicants (Nilsson et al., 2012; Zhang et al., 2014); and initial assessment of the epigenome has been analyzed for the effect of maternal aging on oocyte quality (Ge et al., 2015). But, the effect of differential DNA methylation or histone modifications on the OR during the reproductive lifespan remains to be analyzed. A related issue that is also open for more extensive study is the effect and interaction of the more universal aging process with the uniquely partially programmed aging of the OR reviewed here.

The major challenge in understanding human reproduction for clinical use is the identification of reliable markers of the OR. Today AMH and AFC are routinely used to assess the size of the OR. A recent genome-wide association study has for the first time taken follicle numbers into consideration and found associations with menopause, identifying a variant in *MCM8*, a DNA damage response gene, as a significant effector (Schuh-Huerta et al., 2012). However, again more studies are needed to find direct regulators of the OR that can be used in the clinic to help in family planning. Notably, many potentially useful targets for pharmacological intervention could provide modalities either for birth control or for the augmentation of fertility.

### Conflict of Interest Statement

The authors declare that the research was conducted in the absence of any commercial or financial relationships that could be construed as a potential conflict of interest.
